# Musicdrops@work: Impact of Shared Listening to Short Live Music Interventions on Sense of Belonging and Subjective Wellbeing at Work

**DOI:** 10.3389/fpsyg.2022.865938

**Published:** 2022-04-15

**Authors:** Angelika Güsewell, Sarah Gay-Balmaz, Catherine Imseng

**Affiliations:** HEMU – Haute Ecole de Musique, HES-SO University of Applied Sciences and Arts of Western Switzerland, Delémont, Switzerland

**Keywords:** live music, belonging, wellbeing, workplace, musicking, intervention research, shared listening

## Abstract

Assuming live music can foster belonging in the workplace, this study linked companies in the secondary and tertiary sectors with the world of music performance. Specifically, students from a Swiss music university offered live mini-concerts (10 min of classical music) on the premises of three companies over a period of 3 months. To analyze the impact of these brief musical interventions on the sense of belonging of staff in these companies, a mixed methods approach was adopted using a standardized questionnaire (Barcelona Music Reward Questionnaire, a short online questionnaire on the appreciation of the music and the emotional state induced, and focus groups interviews at the end of the experiment). The short concerts were much appreciated. On the individual level, they led to a greater sense of pleasure and were perceived as a break, a possibility to connect to one’s emotions and above all, as a “moment for oneself.” On the group level, the short concerts allowed the members of teams to meet, prompted conversations and new ways of sharing, created links, and offered opportunities to get to know work colleagues differently and to discover them on a more personal level.

## Introduction

Positive relationships are one of the five pillars of authentic wellbeing identified by Seligman (2011). For Reis and Gable (2003), relationships may be the most important source of life satisfaction and wellbeing. These important relationships include not only family and personal friends but also the wider groups and communities to which we belong. Thus, forming connections and a sense of community with co-workers around a shared mission or identity is a major contributing factor in individuals’ wellbeing and achievement at work.

In 2020, belonging at work became an increasingly popular topic of conversation for HR and business leaders. In fact, it ranked as a top topic in the Deloitte (2020) Global Human Capital Trends survey, with 79% of organizations considering it important for their success. A growing body of work explores the experience of belonging in the workplace, including the conditions and factors favoring its emergence and their respective contribution to strengthening it. The question this paper addresses and which, to our knowledge, is unexplored so far, concerns the potential contribution of music—more specifically of short live music interventions—to the sense of belonging in the context of work.

### Belonging, a Fundamental Human Need

There is general agreement in the literature that belonging—the subjective feeling of deep connection with social groups, physical places, and individual and collective experiences—is a fundamental human need that almost all people seek to satisfy (Maslow, 1954, 1968; Baumeister and Leary, 1995; Deci and Ryan, 2000; Leary and Kelly, 2009; Allen et al., 2021). According to Maslow’s (1968), of the five levels, the need for love and belonging ranks third, after the physiological and safety needs (i.e., basic needs) and is a vital step on the journey to self-actualization. Baumeister and Leary (1995), among the top-cited authors for the concept of belonging, assemble a large body of empirical findings and conclude that “human beings are fundamentally and pervasively motivated by a need to belong, that is, by a strong desire to form and maintain enduring interpersonal attachments. People seek frequent, affectively positive interactions within the context of long-term, caring relationships” (p. 522). More recently, Walton and Brady (2017) define belonging as “a feeling of being accepted, included, respected in, and contributing to a setting, or anticipating the likelihood of developing this feeling” (p. 272). This understanding of the concept brings to light an interesting nuance: a sense of belonging may be felt even in settings where strong relationships do not yet exist, whereas even in settings where close relationships do exist, the feeling may be one of a lack of belonging.

So, why is belonging important? Well, it increases positive emotions like elation, contentment, and calm (Baumeister and Leary, 1995). A sense of belonging also predicts the extent to which individuals find their life meaningful (Lambert et al., 2013). Positive emotions, relationships, and meaning allow thriving (Frederickson, 2009) and are pillars of flourishing and wellbeing (Seligman, 2011). In psychologist Christopher Peterson’s words, *other people matter* (Park et al., 2013). In fact, they matter so much, that they become a source of our self-esteem (Tobin et al., 2014). Self-concept is based on not only one’s unique traits and characteristics (the individual self), but also the attachments formed with significant others (the relational self) and the social groups with which one identifies (the collective self), forming a continuous back and forth between “I” and “we” (Brewer and Gardner, 1996).

### Importance of Belonging at Workplace

Positive interactions in the workplace have been shown to improve job satisfaction and positively influence staff turnover, as employees who experience support from colleagues are more likely to remain in an organization in the long term (Hodson, 2004; Moynihan and Pandey, 2008). Social interactions in the workplace have been found to increase self-reported positive feelings at the end of the workday (Nolan and Küpers, 2009). Furthermore, they have a positive impact on employee engagement, which in turn results in improved productivity or performance outcomes, lower staff turnover and absenteeism, and fewer safety incidents (Gallup, 2015).

Following the experience of remote working during the COVID-19 pandemic, fostering employee belonging appears to be one of the most important human resource issues for 2022. “There is simply no question that the establishment of a culture of belonging must be a strategic point of focus for every business and HR leader seeking an engaged, satisfied, and resilient workforce” [Achievers Workforce Institute (AWI), 2021, p. 3]. In the 2020 Deloitte Human Capital Trends report with its 8,949 global participants, more than 90% of respondents agreed that belonging impacts performance, 79% said that fostering a sense of belonging in the workforce would be important to their organization’s success in the next 12–18 months, and 93% agreed that a sense of belonging drives organizational performance. The findings of the 2020 BetterUp “Belonging at Work study” tend toward the same direction. According to the 1,789 people it surveyed, the benefits of a strong sense of belonging are a 50% reduction in turnover rates, a 56% increase in overall job performance, and a 75% reduction in employee sick days.

### Music as Means of Fostering Belonging

Music is a powerful social magnet, even if one may play or listen to it alone, in the privacy of one’s four walls or with headphones. In almost all cultures globally, and throughout history, music is a social activity (Nettl, 2000) that involves movement to rhythmic sound and plays a significant role in both creating social bonds (Freeman et al., 2000; Dunbar, 2004; Trevarthen, 2012) and permitting meaningful cooperative relationships between groups (Hagen and Bryant, 2003). This effect of musical activity on “social bonding” (the psychological experience of increased social closeness, reflected in prosocial behaviors) may be responsible for the widespread occurrence of musical activities and may have played an important role in the evolution of human sociality (Dunbar, 2012).

In phase with this idea of music creating social bonds, Small (1999) argues for introducing a new word to the English dictionary, that is, “musicking” (from the verb “to music”): any activity involving or related to music performance. According to his own definition, “the essence of music lies not in musical works but in taking part in performance, in social action. Music is thus not so much a noun as a verb, “to music.” To music is to take part in any capacity in a musical performance, and the meaning of musicking lies in the relationships that are established between the participants by the performance” (p. 9). A musical performance is therefore an encounter among human beings wherein meaning is generated beyond the simple meanings assumed to be borne by a musical work. Furthermore, a musical performance is situated; it takes place within a physical and social space “which makes its own meanings” (Small, 1999, p. 13) and which must therefore be taken into account when observing, analyzing, or trying to understand it.

In a similar vein, Moran (2014) examines the social implications identified in embodied music cognition research. This line of thinking comes hand-in-hand with a social interpretation of music, focusing on the real-world basis of its performance, and fostering an empirical approach to musician movement regarding the communicative function and potential of those movements. This paradigm is in contrast to the “individualist” approach, which treats performers and listeners as separable from each other and from the musical stimulus, potentially understating the extent of the interaction among these three components. An essential feature of musical activities is the importance of shared rhythms and the frequent externalization of predictable rhythms (e.g., shuffling feet or swaying heads) that allow synchronization to occur between two or more people (e.g., Bispham, 2006; Merker et al., 2009; Launay et al., 2013). This mechanism of interpersonal synchrony is known as *self-other merging* and may account for the social bonding effect of music. There is evidence that synchronization among people can influence their subsequent positive social feelings toward one another. This has been demonstrated in a number of experimental studies, involving participants tapping synchronously with an experimenter (Hove and Risen, 2009; Valdesolo and DeSteno, 2011), walking in time with other people (Wiltermuth and Heath, 2009) and dancing together (Reddish et al., 2013).

Given the reliance of the self-other merging account of social bonding on simultaneous, similar movements, it is likely that this mechanism does not provide a complete account for the bonding that arises in large group situations. Hence, there is a need to examine the roles played by other factors, for example, endorphins that play a central role in the maintenance of non-sexual, non-kinship social bonds (Machin and Dunbar, 2011) and that arguably mediate the pleasure experienced when listening to music (e.g., Koelsch, 2010).

### Aims and Research Questions

The impact of music listening in work settings has been studied since the 1920s and is well documented (Gatewood, 1921; Antrim, 1943; Kaplan and Nettel, 1948; Uhrbrock, 1961; Fox, 1971; Thorsén, 1989; Lanza, 1994; Oldham et al., 1995; Sloboda et al., 2001; Korczynski, 2003; Lesiuk, 2005; Prichard et al., 2007; Haake, 2010, 2011; Raglio et al., 2020). Nevertheless, these studies and publications focus mainly on job performance, productivity, stress reduction or mood regulation, and concern listening to recorded music.

What about the subjective experience of employees engaging in music at work, what about the impact of different ways of musicking on both a workplace community and individuals? Little research has been done on the subject so far. One exploratory study examined how choral singing at work was experienced and how it changed organizations (Jansson and Balsnes, 2015). Four axes of impact were identified: enjoyment, comfort zone, communality, and identity and roles. According to the authors, singing interventions at work can change the way how colleagues view each other and transform the workplace as a practice community. The present research is a continuation of this exploratory study, while proposing another form of musical engagement at work, namely collective listening of live music. The objective was to provide answers to the following main research question: How is a series of short live music interventions offered during work time experienced individually and collectively, and in what way does this experience foster a sense of belonging among the staff who attended? A secondary research question sought to identify factors—for example, the composition of teams, the structure or culture of the company, or the infrastructure available—facilitating or hindering the impact of the short live music interventions on social bonding and hence on the feeling of “togetherness.”

## Materials and Methods

### Participants

Three companies in the canton of Vaud (Switzerland) took part in the project: a car garage, an engineering office, and a kitchen sales space. A series of short concerts was offered to them free of charge. In exchange, the companies agreed to free up some or all of their staff for 10 min a week outside their usual breaks to attend these musical moments, to allow data to be collected through questionnaires, interviews, and participant observation, and to host a piano for the duration of the project (and to cover the costs associated).

Table 1 gives some indication of the teams from each of the three companies part in the project (number of staff, socio-demographics, and music listening habits). The kitchen sales team appears to strike the best gender balance, followed by the engineering office team, whereas the composition of the garage team was strictly male (only 25 of the 64 car garage employees filled out the preliminary questionnaire, mainly due to communication and coordination problems between the HR department—responsible for distributing and collecting the forms—and the team, but this had no impact on the gender distribution). On average, the teams of the engineering office and of the car garage were younger than the kitchen sales team. In terms of educational background, the engineering office had the highest, but also the most diverse level of education.

**Table 1:** Socio-demographic data of the staff who participated in the project.

	Car repair		Kitchen sales		Engineering office	
	Workshop staff, *N* = 64		Office team, *N* = 30		Whole staff, N = 33	
	*N*	Valid %	*N*	Valid %	*N*	Valid %
**Gender**						
Men	25	100.00	17	58.6	22	68.8
Women			12	41.4	10	31.2
**Education**						
CompulsorY	7	29.2	1	3.4	3	9.4
Apprenticeship	16	66.7	16	55.2	12	37.5
Baccalaureate	1	4.2	6	20.7	2	6.3
Bachelor			2	6.9	2	6.3
Master			4	13.8	10	31.3
PhD					3	9.4
**Music listening**						
<1 h/week	1	4.0	1	3.3	1	3.2
1 h/week	3	12.0	4	13.3	6	19.4
3–6 h/week	7	28.0	10	33.3	9	29.0
> 6 h/week	14	56.0	15	50.0	15	48.4
**Musical practice**						
Past	9	36.0	17	56.7	14	45.2
Current	1	4.0	7	23.3	6	19.4
Styles of music listened to	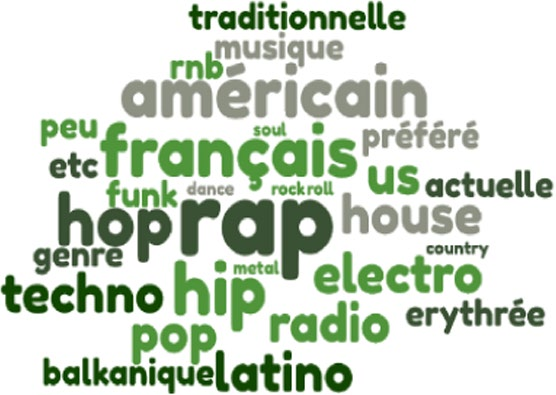		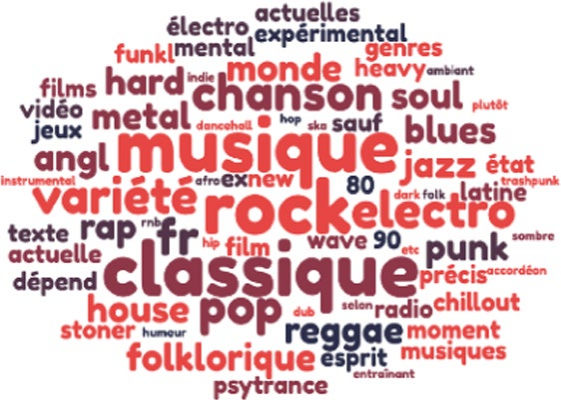		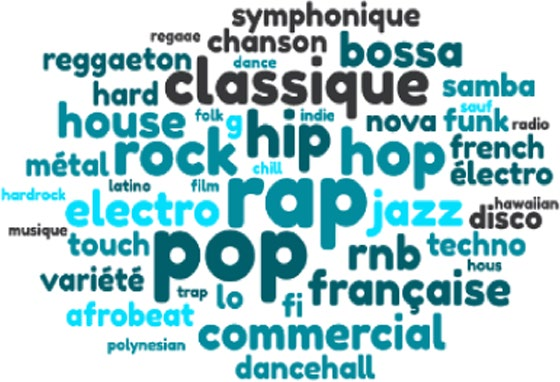	

*The category of staff and the total number of persons concerned by the project are, for each of the three companies, followed by the answers of those who filled in the French version of the Barcelona Music Reward Questionnaire (BMRQ;* Saliba et al., *2016**). Each time, the N and % of valid answers are indicated*.

There were no significant differences in terms of hours of weekly music listening. In terms of musical practice—present and past—the kitchen sales team came out on top, followed by the engineers, then the garage employees. Finally, with regard to the styles and genres of music listened to by the employees of the three companies, the repertoire evoked by the kitchen sales team is more varied than that of the engineers, who in turn evoked a wider range than the technicians and mechanics of the garage.

### Method

**Figure 1:**
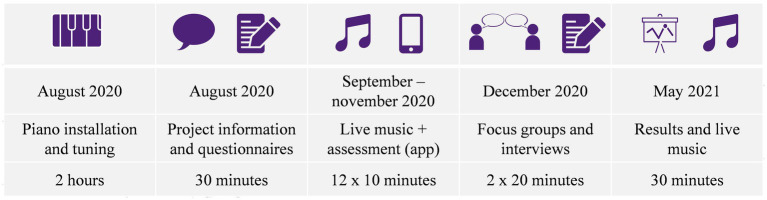
Calender of the project.

Separate agreements specifying the terms and conditions and above all the general timetable for the project (Figure 1) were established with each company. However, the timetables agreed at that stage were later modified due to the COVID-19 pandemic. The start of the weekly musical interventions, planned for March 2020, was postponed until mid-August. In addition, instead of the 12–16 mini-concerts initially planned, only 11 consecutive concerts could be performed before the arrival of the second wave of coronavirus infections in Switzerland and the increasingly severe restrictions introduced as of the end of November 2020.

Once the agreements were signed, professional music students^1^ ready to play a short program (6–8 min of music) in each of the three companies were recruited. Based on their proposals, a varied repertoire (always in a small formations, duo, or trio), mainly classical, was put together (see Supplementary Table).

To gather information on how these interventions were received and experienced, a mixed methodology was used combining questionnaires, observations, and interviews. Before the first concert, all collaborators involved in the project were invited to complete the French version of the Barcelona Music Reward Questionnaire (BMRQ; Saliba et al., 2016) with a view to obtaining select socio-demographic information and to understanding the place of music in their lives. The BMRQ examines five main facets that characterize musical reward experience in individuals: musical seeking, emotion evocation, mood regulation, social reward, and sensory motor. Musical seeking refers to the way individuals pursue music-related activities (e.g., attending concerts, playing an instrument) or seek additional information about music they listen to (performers, composers). The emotion evocation aspect is related to the emotional impact of music on individuals. In contrast, the ability of listeners to use music to modulate their emotions (i.e., to relieve stress, to release emotions, to comfort) is referred to as mood regulation. The social reward facet examines the social bonding effect of music on individuals. Lastly, the sensory motor facet addresses the capacity of music to induce body movements in certain individuals (i.e., head nodding, dancing). The questionnaire contains 20 statements, four per facet. Participants indicate the level of agreement with each statement by using a five-point scale ranging from “fully disagree” (1) to “fully agree” (5).

During the musical moments, participant observation (logbook and observation grid) allowed the course of the interventions to be recorded. In addition, after each concert, listeners used an application downloaded to their mobile phones to rate the following aspects on a five-point scale ranging from “fully disagree” (1) to “fully agree” (5): their appreciation of the music performed (I enjoyed the music that was played), their emotional experience (I was in a constant mood during the musical intervention), their physical reactions (My body was reacting during the musical intervention), their feeling of living a common experience (I felt close to the other people present), their desire to share about this experience (I wanted to share with the other people present), and their ability to leave or forget the reality (I forgot the realities of my immediate environment). Finally, and after the series of musical interventions, four focus group interviews were held with 17 volunteer staff members from each of the three companies in order to explore with them further specific issues relating to the experience. Taking part in the first interview were four long-standing employees of the car garage (11.9 years on average in the company); in the second were two women and three men of the kitchen sales team (5.3 years on average); in the third, one woman and three young men working in the engineering office (3.5 years on average); and in the fourth, four more-experienced men from the engineering office (5.3 years on average). Each focus group interview lasted 30–45 min.

### Data Analysis

The responses to the preliminary questionnaire (BMRQ; Saliba et al., 2016) and the data from the smartphone application (i.e., feedback on participants’ subjective experience of interventions) were imported into SPSS (version 24) for simple statistical processing (descriptive statistics, e.g., frequencies and means; Spearman correlations, *t*-tests). The interviews were recorded, transcribed in full, and then coded using NVIVO software, in order to carry out a thematic analysis. Three successive deductive coding steps were carried out in NVIVO, first identifying “parent nodes” reflecting the main categories of analysis, and then subdividing them into child themes and sub-nodes, some of which were further sub-divided into grandchild sub-nodes.

### Ethical Concerns

To respect confidentiality, both of staff and management, the names of the three companies that participated in the project do not appear in this publication, and the names of the focus group participants have been changed. To allow employees to express themselves freely, it was agreed with HR managers and management that only aggregated data would be communicated to them. Finally, in accordance with our data management plan, the raw data made available on the SWISSUbase platform^2^ have been anonymized.

## Results

Relying on the focus group interviews, we will first examine how the series of short live music interventions was experienced at the time of listening. We will then look at its impact on the lives of the teams and therefore on its possible contribution to the feeling of belonging of the employees. Finally, following a question posed by Small (1999), that is, “[w]hat does it mean when this performance takes place at this time, in this place, with these people taking part?” (p. 13), we will try to identify factors likely to favor the impact of the musical interventions.

### Experience at the Time of Listening

At the individual level, the mini-concerts seem to have been experienced as moments of pause and relaxation.

You feel better afterwards! In any case, speaking for myself, I did, it was… Yeah, I felt, I felt good, almost as rested as after a long break, when actually it was only 10 min [Lisa].

This pause provided a breath of fresh air (an idea mentioned several times) and contributed to wellbeing, in the moment, sometimes also for the rest of the day:

I think it went with me a little for the rest of the day… We were a bit tense, we came to listen to the music, we got some fresh air, and then back off to work again and, I do not know, there was a bit, a bit of a calming side to it, well … It was… I liked it, well precisely when there were music days, then I knew that at 10 o’clock I was going to enjoy it and that the rest of the day was going to go well: there’s a bit of an effect that sets us up to be calm, well, in a state of well-being for the whole day [Leonie].

The concerts made it possible to leave, to escape, to travel in thought:

It also lets you quickly imagine yourself somewhere far away, or to think about something else, to escape, for at least 10 min of the eight and half hours you spend at work. Even if it’s not the music I usually listen to, it already allows me to escape a bit [Andrew].

“Getting away” at the concerts allowed the employees not only to distance themselves from their tasks and immediate environment—“Some kind of calm moment when you could imagine some other place while the music played but that you could also see” [Noah]—but also to be aware of their emotions and the associated bodily sensations, as illustrated by the following interview extract:

Nelly. Yeah, it was very strong … I was moved the first time.

Leonie. Yeah, it makes you shiver.

Nelly. I was shivering and almost had tears in my eyes.

Listening to the music, observing the musicians and their movements, and feeling their emotions were contagious and also invited movement:

I mean the emotions of the musicians I mean it was moving and it almost made us want to move too so you really feel … the music makes you travel. It’s a good way to let off steam and then … [Leonie].

The difference between the impact and the experience of a “canned” and live music was noted: “I think that … there’s more feeling when it’s real instruments and even more when you see the people in front of you, there’s more connection, it’s true that you can notice it” [Josiane].

Finally, listening to the music was a moment that some employees wanted to keep and live for themselves.

Often I close my eyes because I do not want the reaction of someone next to me to have an impact on what I am thinking. So eyes closed and I live for myself, I do not care about what’s going on around me (laughs) … I do not want the outside world to interfere with what I’m thinking [Pauline].

They talk about it with some embarrassment, as this attitude might be perceived as selfish: “I’m very selfish, uh, I do not share with anyone, these moments I lived only for me and with me” [Olivia].

Although the listening is primarily personal and participants have strategies to place themselves in their own bubble, the concerts take place in the workplace. It is not, therefore, possible for them to ignore the reactions of colleagues completely:

Exactly, and then you see a colleague tapping with his leg or doing this, and you think, ‘oh, he’s getting into it’, I would not have thought of that, and then, yeah … But on a more personal level, it was very rare that I actually looked at my, my colleagues to see what reaction they had [Lisa].

And it is precisely this awareness of a commonly felt experience that brings us to the second section of the results section, the one that focuses on the impact of the musicdrops@work intervention on the lives of the teams.

### Impact on the Lives of the Teams

Several participants noted that attending the musical interventions together created a moment of convergence and assembly during the working day, whereas in everyday life not everyone necessarily interacts with everyone else, especially in times of pandemic:

It allowed us all to see each other for a little while during the day, when normally, well, we do not meet, well, sure, there are breaks etc., but we do not always all take our breaks at the same time … And nowadays, with the restrictions, there cannot be more than a few people in the canteen and so on … So it’s true that it meant that there were little moments when we could all see each other a little bit during the day, when on some days we do not even cross paths [Lisa].

Listening to the same music, sharing the same feelings was experienced as unifying for the team or group:

Lisa. It brings us all together at some point, all in the same … in the same place to listen to the same thing, to have the same feeling really … Yeah, I think it’s somewhat …

Alan. It creates a feeling of togetherness.

Lisa. Yeah, it brings the team together.

Attending a concert with one’s colleagues arouses curiosity: How will they react in this new, somewhat incongruous situation? What will happen?

We watch or listen to music in specific places, in theatres, festivals, etc. In headphones too. But not at work, not in an office. So there was also this little bit of a different side to it, which nudged up the level of curiosity about seeing what a group performance would be like in an office [Andrew].

The conversations that the moments of musical listening kindled were different from ordinary professional exchanges and created a more personal contact:

Afterwards in the office, I think it leads to chat, the staff coming together where we all meet up and listen to music, create connections or … or share uh, emotions and so on. Even if it’s a short moment, a short period of time. It was enough to let you feel something and then either share or not share, keep it to yourself, that is … After all, everyone reacts differently [Andrew].

Discussions and sharing covered a range of topics, for example, whether the music played on that day had been liked or not, the sensations or emotions evoked, the personal experiences with music or with playing an instrument, recommendations for other concerts.

Well, I think obviously, because there’s this concert, we talk about it afterwards, and that allows us to share, to … to share feelings, if we liked it, if we had already heard, listened to, talked about music: ah there’s a concert, ah yes, great [Theo].

Generally, these moments of discussion were not long:

William. Yeah, straight after the concert, the mini-concert we said ah yeah, that was good, I liked it and then, then it was back to work again.

Noah. I agree, 5, 10 min of chat no more and then we went back to the usual routine.

… and they seem to have taken place mainly between those who already knew each other well or who worked side by side. The common experience therefore seems to have strengthened pre-existing links rather than created new ones:

After the concert there was a little opening up between … between the closest colleagues etc. So the break, let us call it an interruption, also went on a bit longer with discussions, chatting, uh. The break went on a little longer. The smokers go to the balcony and there they are talking about it, they chat a little … Anyone left around the islands [groups of work tables] as we are split into groups of four, among the four we also spoke a little bit about – about the concert so I think yeah [Alan].

Arising often was that the exchanges around the music increased the acquaintanceship of certain colleagues, on a more personal level, and sometimes uncovered totally unexpected facets of their personality, of their lives, as the following brief exchange illustrates.

Olivia. We found out that our colleague played the oboe and we were stunned (laughs) Guillaume! He used to play when he was young, but then it was a bit of a shock to find out.

Arthur. Yeah, then we talk …

Olivia. Because it’s not at all the guy’s image, I cannot even imagine him playing music! Guillaume driving a tractor or a forklift, no problem, driving heavy machinery. But playing an oboe? I’m sorry, but it was a shock …

Gregory. Oh yeah there was a very strong contrast (laughs)

Arthur. I think these contrasts are really cool and it’s really interesting to get to know a bit more about someone you work with.

The presence of the pianos at the companies for three months, clearly visible and present, also led to discoveries …

Yeah, then perhaps it also means we find out something unexpected about someone, or at least something that we did not know about them! We have little … It’s true that … it’s not easy to get under someone’s skin I mean, yeah, I would not go so far as to say to get under their skin, but to get to know them a little better on a personal level, and then I think that it was also quite positive because it’s true that then suddenly one of our colleagues started playing the piano. She played a little bit of Amélie Poulain and it was really cool. And we did not necessarily expect that from her! [Alan].

…. so much so that the kitchen sales team even suggested that a piano instead of the planned table football would be a plus for office life:

Lisa. So it’s true that it … we think if we had a piano in the long term instead of table football and whatever, maybe it’s something that … that would bring in well …

Mia. Warmth.

### Factors Influencing the Impact of Music Interventions

A first factor which appears to be related to both the experience of the musical interventions and the exchanges that followed is the composition of the teams of the three companies that took part in the project. We have already seen above that they differed in respect of gender distribution, the level of training of employees, their personal musical practice, and their tastes and preferences in terms of the styles and genres to which they listened. Looking at associations between socio-demographics and the five BMRQ facets, respectively the data from the smartphone application (Table 2) shows that men scored significantly higher than women on the BMRQ sensory motor facet [*t*(34) = 3.52, *p* = 0.000]. Those who received musical and/or instrumental training during their childhood or adolescence scored significantly higher on the BMRQ emotion evocation [*t*(34) = 2.95, *p* = 0.006], mood regulation [*t*(34) = 2.75, *p* = 0.010], and social reward facets [*t*(34) = 4.68, *p* = 0.000]. Furthermore, they reported on more bodily reactions [*t*(33) = 2.45, *p* = 0.020], and on greater appreciation of the short concerts [*t*(33) = 3.45, *p* = 0.002] than those who never received musical training. Higher education, in turn, was correlated significantly with the BMRQ sensory motor [*r*(33) = 0.36, *p* = 0.035] and social reward facets [*r*(33) = 0.38, *p* = 0.032] on the one hand, with experiencing bodily reactions [*r*(32) = 0.36, *p* = 0.038] on the other. Finally, using music for mood regulation was correlated positively with the number of weekly hours of music listening [*r*(34) = 0.36, *p* = 0.031].

**Table 2:** Descriptive statistics, reliabilities, of the BMRQ facets and the application scales, and associations with socio-demographic variables.

	Descriptive statistics						Socio-demographics						
									*T*-Tests		Correlations^a^		
	*M*	*SD*	*S*	*K*	Min	Max	Sex		Training		Edu	Listen	Age
							*t*	*p*	*t*	*p*			
**BMRQ facets**													
Musik seeking	3.75	0.84	−0.42	0.59	1.33	5.00	−0.42	0.679	1.89	0.067	0.16	0.21	−0.21
Emotion evocation	3.81	0.77	−0.29	−0.84	2.25	5.00	1.03	0.310	2.95	0.006	0.30	0.02	0.28
Mood regulation	3.85	0.82	−0.54	−0.54	2.00	5.00	0.21	0.834	2.75	0.010	0.19	0.36^*^	−0.30
Sensory motor	3.60	0.85	−0.18	−0.64	1.75	5.00	3.52	0.001	0.922	0.363	0.36^*^	0.08	−0.24
Social reward	3.16	0.86	0.09	−0.20	1.25	5.00	1.06	0.295	4.68	0.000	0.38^*^	0.24	−0.04
**Smartphone application**													
Consistent mood	4.18	0.51	−0.11	0.45	3.00	5.00	−0.90	0.377	−0.82	0.419	−0.16	0.15	0.17
Bodily response	4.06	0.53	0.00	0.17	3.00	5.00	1.07	0.292	2.45	0.020	0.36^*^	0.16	0.01
Feeling close	3.74	0.64	0.53	−0.78	3.00	5.00	−0.87	0.931	0.62	0.543	0.21	0.23	0.04
Forgetting reality	4.01	0.54	0.17	0.07	3.00	5.00	1.33	0.193	0.77	0.443	0.32	−0.10	0.09
Desire to share	3.78	0.69	0.48	−1.01	3.00	5.00	0.22	0.831	1.60	0.118	0.24	0.17	0.23
Enjoyment of music	4.50	0.49	−0.83	0.64	3.00	5.00	1.12	0.271	3.45	0.002	0.24	0.28	0.06

*N = 36 (male = 24, female = 12). S = skewness, *K* = kurtosis. Sex (1 = male; 2 = female), Training = musical training (1 = no; 2 = yes), Edu = educational level (1 = compulsory education; 2 = apprenticeship; 3 = baccalaureate; 4 = bachelor; 5 = Master; 6 = PhD), Listen = weekly music listening (1 = less 1 h/week, 2 = 1–3 h/week; 3 = 3–6 h/week; 4 = > 6 h/week)*.

a *Spearman correlation*.

* *p < 0.05*.

The fact of having had musical training (or not) thus seems to have played an important role in the experience of musical interventions. The impact of the project was not the same in teams with musically active people as in those without. An excerpt from one of the interviews conducted in the engineering office points in this direction: the employees interviewed mention the fact that there are several amateur musicians in their team who seized the opportunity to discuss this “personal side” and took great pleasure in it:

It seems to me there’s as many as 5–6 musicians here in the office, even 6–7, well whatever their levels, yeah, musicians tho’. And it’s pretty cool to, to talk about that, then to get to know a bit about the personal side of a colleague and then yeah … It’s cool to connect in these … moments [Andrew].

A second factor that seems to have played a role is the hierarchical structure of the companies—ranging from rather horizontal in the engineering office to very vertical in the garage—that is, the trust of the employees toward their management and especially their perception of the reasons that may have led them to take part in the project. While the employees in the engineering office knew well their boss and his affinity for music, especially classical music, which was a sufficient explanation for participation, the workers in the car repair shop expressed distrust:

*Interviewer. And why do you think your company decided to participate in this project?*

William. No idea … (laughs)

Chris. We see evil everywhere, there’s surely a … (laughs)

William. Because it was organised with HR

Chris. There! You should know HR is not part of our world

*Interviewer. It’s two separate worlds?*

Chris. Yes, it’s two separate companies, in fact

*Interviewer. Oh, really …*

Chris. Yes. No, HR used to be part of the company, now it’s an independent external firm that belongs still to the same group, but we hardly know them anymore, to be honest.

William. They are not allowed to be buddies with the employees.

A third factor that often came up in the interviews is the degree of autonomy that employees enjoy in organizing their work, and the nature of their task. For the engineers, it seems to have been easy to organize themselves:

Gregory. In September–October I was working on something due in October and I said to myself well I’ll take 10 min – a quarter of an hour for that and too bad I’ll stay on in the evening when I do stay longer, but I absolutely wanted to have that … that interruption. I wanted to come absolutely.

Theo. In our industry we can manage our … our work quite easily and when we cannot, well, we just do not come.

Leonie. It’s not the time, 10 min, it’s not what influences our rate of professional activity, well … it’s not the 10 min … that necessarily bothers us, I’d say.

The forced break was experienced as more complicated and stressful for the mechanics and technicians in the garage:

William. Well it all depends on what work we are doing. There are times when you cannot stop. So in painting, if we were spraying, well, doing a spray job, we would not come. Or we’d finish a coat, we’d come and listen to a bit and then we’d have to go back and continue …

Antonio. Yeah, it was cut short then but … then, what’s for sure is that when we have a schedule, the cars have to be ready! Especially in mechanics, the cars come in for a service, the customer can come and pick it up at 3 pm, sometimes 4 pm, sometimes 5 pm. So uh it’s true that when we are really busy in terms of our schedule, sometimes a quarter of an hour, well that’s all it takes, we can really need it.

**Figure 2:**
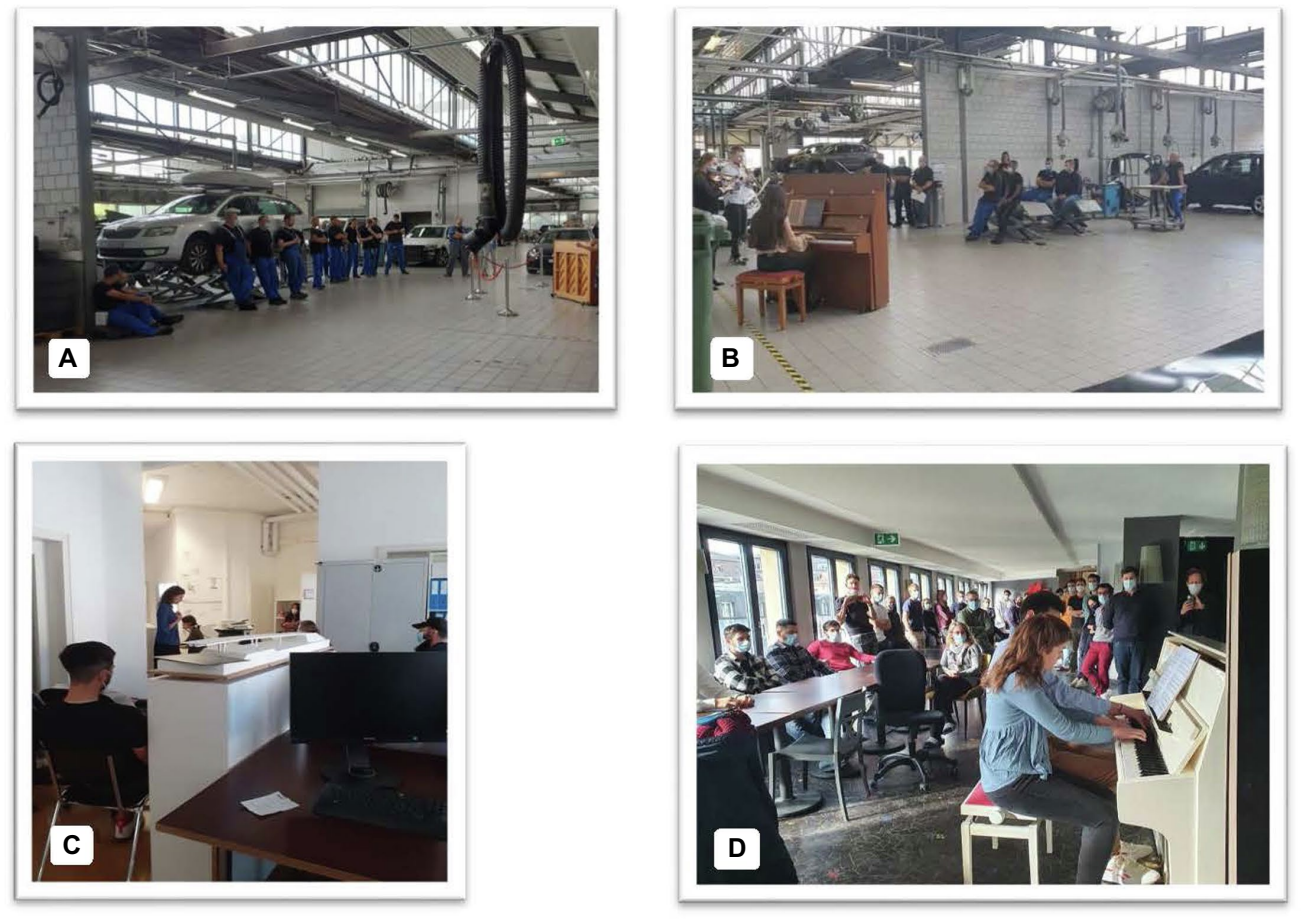
Ritualization. Car garage, first **(A)** and last **(B)** concert; Engineering office, fourth **(C)** and ninth **(D)** concert.

Integrating a new practice into the professional context—in this case, offering employees the opportunity to interrupt their work to listen to live music or concerts—required ritualizing these moments, defining times and a dedicated space for the interventions, and it is a fourth factor which seems to have played a role. In all the companies, the space around the piano was set up in order to create a concert setting, a kind of “stage.” A certain distance between artists and audience was naturally created. However, there was not necessarily any seating for the audience, who therefore remained standing during the first concerts. After a few weeks, the car garage employees organized themselves, appropriated elements of their work environment to settle down more comfortably, as they would do on seats during a concert in a more traditional setting (see Figures 2A,B).

In the engineering office, the piano was first placed in a corridor, which did not allow the whole team to meet. It meant that most collaborators remained seated at their workstations (Figure 2C). The expansion of the premises allowed the piano to be moved after a few weeks to a space adjacent to the offices proper, which allowed the whole team to meet in a more neutral concert space (Figure 2D). According to the staff, moving was crucial, as it reinforced the “exceptional” aspect of the concerts, considered as events with their own space, disconnected from the usual tasks.

Arthur. Yeah, I think at the beginning, we did not think well about the positioning of the, of the performance, a tiny corridor, I think that did not help for the first few shows, so yeah sorry about the first few uh … it was really a small spot and everyone was a bit more scattered. And then when we moved here to the restaurant, uh …. it was much better – it feels to me that there was more movement involved, people really had to get up to come and listen.

Gregory. Location matters

Kevin. Exactly, exactly yeah!

Gregory. It boosts the atmosphere, I do not know, it creates.

A fifth factor is related to the classical repertoire played by the students. It was clearly more familiar and hence more accessible to the teams in kitchen sales and engineering than to the employees in the garage. The idea that the short musical interventions would allow listeners to discover and appreciate a repertoire and style of music unknown to them, was only partially confirmed. Although the post-concert questionnaire showed that the music offered was generally appreciated, several participants, especially in the garage, mentioned the fact that they would have preferred listening to another repertoire, jazz, pop, rock or world music. Accordingly, the audience’s familiarity with the repertoire clearly seems to be a facilitating factor. Also in connection with the choice of repertoire, another point emerged: the fact that the teams would have liked to have been involved, to make proposals and for them to be taken into account.

It’s always nice to have a little break like that in the middle of the workday. Then what’s even better was the day when … I think it was you who played a song that we had asked for, I think that was it. It was great and there was even more of a connection because we related it to … something that many of us know [Kevin].

A sixth and final factor is the perspective gained from talking to customers, family and friends. The fact that they were surprised, did not believe their eyes or ears, and were interested and even enthusiastic allowed the employees a certain pride. This, in turn, had an impact on their experience:

Josiane. I received some comments, yes! People wanted to know how it works etc., I told them roughly what it was, once a week and yes, they found it interesting too, yes.

Cedric. That’s right, it’s true that when we talked about it, people around us were like “oh yeah, you do that at work? Huh! well, that’s not so bad! (laughs).”

## Discussion

### Summary of the Main Results

The main objective of the musicdrops@work project was to offer a series of short weekly musical interventions in three companies in Lausanne and to study the impact of this measure on the subjective experience of staff, on the lives of the teams, and thus on the sense of belonging. A second objective was to improve our understanding of the conditions that facilitated or hindered the insertion and reception of these mini-concerts within the three companies.

The level of appreciation of the interventions and of the music played was globally high across the three companies. The teams’ greater or lesser familiarity with classical music does not seem to have played a role in this dimension of subjective experience. In all the companies, the staff considered the concerts to be a moment of “relaxation” that allowed them to create distance from the tasks at hand and their immediate environment, co-workers included. These moments were rather savored by each for themselves, in a somewhat “selfish” way as some put it, so as not to be influenced by the reactions of others. The musical interventions therefore allowed a form of self-empowerment as defined by Hatzfeld (2002), an opportunity to “reacquire a form of autonomy within the constrained framework of work,” notably through imagination. Thus, the concept of musical affordance (DeNora, 2001), according to which the listener appropriates the music according to his or her needs at the time of individual listening, also seems to apply in the context of group listening during a work break. The mini-concerts were an invitation that employees could seize and a resource that they could mobilize in order to regulate their affects and act on their wellbeing.

At the group level, the avenues explored in the introduction concerning the possible impacts of collective listening on the group were confirmed. The notion of “connection [*lit*. sharing]” was often mentioned by participants during the focus groups. They emphasized that the moment of group listening was perceived as moment of convergence and assembly, as a shared experience (even if lived individually) outside of their professional tasks. It not only lead to new exchanges—concerning the appreciation of music, feelings, and emotions evoked, personal experiences with music or with playing an instrument—but also allowed them to get to know some co-workers better and to discover previously unknown facets of their lives or personalities. It appeared that these exchanges and discussions mainly took place between those who already knew each other, reinforcing pre-existing relationships. This finding is consistent with what we know from the literature, namely that the impact of collective listening depends primarily on the quality of the relationships between listeners, but that it can also modify pre-existing relationships or enable new ones (Hargreaves and North, 1999; Groarke and Hogan, 2015). In summary, both the literature and our results indicate that group listening fosters positive intra-team dynamics and can therefore contribute to community wellbeing.

Even if the exchanges and interactions generated by the music interventions were occasional, as several participants in the focus groups pointed out, they seem to have had an effect on team dynamics. The concept of “high-quality connections” (Dutton and Heaphy, 2003), short, subjectively positive experiences between two people that generate beneficial outcomes, could explain the impact of these rather time-limited interactions. High-quality connections are marked by three subjective experiences: feeling alive, positive regard, and mutuality; all of which have benefits for wellbeing. High-quality connections can happen between two total strangers (e.g., exchanging a sincere smile with someone you see also buying diapers in the check-out line) or within established relationships (e.g., having a genuine “good morning” conversation with a co-worker). The mechanisms that influence high-quality connections are cognitive (e.g., other awareness, perspective taking), emotional (e.g., positive emotions, empathy), and behavioral (e.g., respectful engagement; Stephens et al., 2011).

Several conditions and factors favorable to the adoption of musical interventions in companies were identified, for example, the composition of the teams, the company’s management style, the fact that the staff had tasks that allow them a certain flexibility and autonomy in the organization of their work and time, but also the fact that the concerts took place in a dedicated space, which contributed to creating a ritual away from their workstations. This is in line with Small’s idea of a musical performance as a situated experience taking place within a physical and a social space “which makes its own meanings” (Small, 1999, p. 13) and which must therefore be taken into account when observing or analyzing or trying to understand it.

In all three companies, the decision to host the musicdrops@work project was “top-down.” Even in the engineering office, whose management was less traditional and vertical than in the other two companies, the teams were not involved in the organization and implementation of the series of short concerts (choice of location for the piano and the “stage,” choice of timetable, choice of repertoire, etc.). This observation raises the question of whether the musical interventions—beyond promoting subjective wellbeing and belonging at work through the appropriation of music as a tool for regulating subjective affects and through the encouragement of interpersonal dynamics—truly constituted a “social innovation” as defined by Cloutier (2003):

[…] a “new response” to a social situation deemed unsatisfactory, a situation that may occur in all sectors of society. Social innovation fits this title because it aims at the well-being of individuals and/or communities. It is defined in action and sustainable change. It aims to develop the individual, the place of life (territory) or the enterprise […] Some researchers also define social innovation by its process. Social innovation is then that which results from the cooperation between a diversity of actors (p. 12).

Although a new practice aimed at “the wellbeing of individuals and/or communities” and “defined in action” has been introduced into companies, it is likely that their one-off nature has not led to “sustainable change.” Furthermore, and centrally, the implementation of the musicdrops@work system was not the result of a “cooperation among a variety of actors,” but of a proposal from outside (the research team) and of a unilateral decision taken internally (management and/or HR manager). It is not clear whether the objective of the latter was indeed to respond to a demand or a need, and hence to improve a “social situation deemed unsatisfactory” (and if so, which one), or whether musicdrops@work was above all a great opportunity to be seized.

### Limitations

As with much intervention research planned for, 2020, musicdrops@work was heavily impacted by the COVID-19 pandemic. As already mentioned, the series of weekly musical interventions in the three companies, initially planned for 12–16 consecutive weeks, began with a five-month delay and had to be stopped after 11 weeks, due to the arrival of the second wave of infections and the new severe restrictions. Furthermore, the program had to be revised several times to take into account the changing health measures and withdrawals of quarantined or ill musicians. Apart from this obvious impact on timing and organizational aspects, the health crisis had a more indirect influence on the project and the results: in Autumn, 2020, very few “normal” live concerts could take place in Switzerland. For the student musicians, the musicdrops@work project was one of the very few opportunities to perform in public. When asked about their experiences, they repeatedly referred to the intensity of the emotion they felt, to the importance of these few minutes for them, to the responsibility they bore, given the exceptional nature of their performance. On the employees’ side, a great intensity in listening was visible and noticeable. Given that the pandemic did not allow for the deployment of the methodological system as initially planned, and that furthermore it reinforced the intensity of the experience of both musicians and listeners, the results of our study must be interpreted with great caution, pending confirmation by a second series of concerts or by a second study that would take place under normal circumstances.

Boumaza and Campana (2007) write that the company is a “difficult” terrain that is not easily “approached” or “tamed,” which requires “a certain pragmatism” and the establishment of “risk avoidance strategies” (p. 5). This is what we experienced: the methodology had to be adapted several times to the reality of the companies. The length of the questionnaires, the time needed to fill them in, and even the language level and/or the content of the envisaged standardized tools (employee experience questions were not welcome in all companies) were all subject to negotiation, and it was necessary to scale down to obtain the cooperation of HR managers or directors. We therefore decided not to include a questionnaire assessing employees’ sense of belonging and opted for a more qualitative approach, which would capture the experience of the teams and the changes in interactions among colleagues. Nevertheless, the use of such a questionnaire should be considered should the experiment be repeated, ideally with a longer series of concerts.

A third limitation of the research concerns the sampling. Although all employees were able (in some cases obliged) to take advantage of the musical interventions, there was no onus or checks on the completion of the questionnaires nor on participating in the interviews: participation and in some cases recruitment were done solely on a voluntary basis. It is therefore possible that only people who were relatively enthusiastic about the music interventions and willing to talk about them came forward, and that this led to a bias by reducing the diversity of views.

### Conclusion

The musicdrops@work project is intervention research aimed at implementing and studying the impact of short live music interventions on belonging at work, and therefore on the wellbeing of individuals and teams. A positive impact of the intervention was highlighted by the observation of the dynamics that developed over the weeks around the mini-concerts, by the data that were collected after each moment of collective listening, and by the analysis of the group interviews conducted at the end of the experience with 17 volunteer employees. This was true both at the individual and group levels. On the former, the mini-concerts were experienced as a moment for oneself, as an opportunity to distance oneself, as an invitation to self-empowerment, and as a resource for acting on one’s wellbeing. On the latter level, the mini-concerts generated exchanges and interactions, and provoked moments of “high-quality connections,” thus contributing to employees’ sense of belonging.

These results lead us to reflect on the place of music and the musician in society, and on their potential in the world of work. A recently published article refers to the musician as a “maker in society” (Gaunt et al., 2021, p.1). According to the authors, “questions […] cannot be ignored about the ways in which musical practises are […] of value in societies and the degrees to which these are realised, the roles musical practises may play within rapidly changing situations, and how they may be part of nurturing flourishing and inclusive societies for the long term. In many ways a contemporary zeitgeist is crying out for the creativity and humanity of music and the arts: their unique potential to uplift, heal, and engage people in expressing themselves, to help make sense of experience and challenge perspectives, and to contribute to building and sustaining communities” (p. 3).

Too little is known about the potential for social innovation to be found in inclusive musical practices that avoid revolving around excellence, elitism, or “star” culture, but instead conceive of music as a social practice and invite participation. It is up to educational and research institutions to seize this opportunity by proposing training programs that place the concept of artistic citizenship at the center of their curricula, by attaching value to the social engagement of their students and graduates, and by promoting research activities that aim simultaneously to change reality (i.e., action or intervention research) and to increase our understanding of the mechanisms that underlie the change.

## Data Availability Statement

The datasets presented in this study can be found in online repositories. The names of the repository/repositories and accession number(s) can be found at: https://www.swissubase.ch/en/catalogue/studies/14067/17102/overview.

## Ethics Statement

Ethical review and approval was not required for the study on human participants in accordance with the local legislation and institutional requirements. The patients/participants provided their written informed consent to participate in this study.

## Author Contributions

All authors listed have made a substantial, direct, and intellectual contribution to the work and approved it for publication.

## Funding

This project was funded by the Swiss National Science Foundation (project number 190782) and by the University of Applied Sciences Western Switzerland.

## Conflict of Interest

The authors declare that the research was conducted in the absence of any commercial or financial relationships that could be construed as a potential conflict of interest.

## Publisher’s Note

All claims expressed in this article are solely those of the authors and do not necessarily represent those of their affiliated organizations, or those of the publisher, the editors and the reviewers. Any product that may be evaluated in this article, or claim that may be made by its manufacturer, is not guaranteed or endorsed by the publisher.

## Footnotes

^1^HEMU – Haute École de Musique.

^2^SWISSUbase is a national cross-disciplinary solution for Swiss universities and other research organizations in need of local institutional data repositories for their researchers. The platform relies on international archiving standards and processes to ensure that data are preserved and accessible in the long-term. To access the musicdrops@work project: https://www.swissubase.ch/en/catalogue/studies/14067/17102/overview.

## References

[ref1] Achievers Workforce Institute (AWI) (2021) Culture report on belonging at work. Available at: https://hrmasia.com/achievers-culture-2/ (Accessed April 01, 2022).

[ref2] Allen K.-A. Kern M. L. Rozek C. S. McInerney D. M. Slavich G. M. (2021). Belonging: a review of conceptual issues, an integrative framework, and directions for future research. Aust. J. Psychol. 73, 87–102. doi: 10.1080/00049530.2021.1883409, PMID: 33958811 PMC8095671

[ref3] Antrim D. K. (1943). Music in industry. Music. Q. 29, 275–290. doi: 10.1093/mq/XXIX.3.275

[ref5] Baumeister R. F. Leary M. R. (1995). The need to belong: desire for interpersonal attachments as a fundamental human motivation. Psychol. Bull. 117, 497–529. doi: 10.1037/0033-2909.117.3.497, PMID: 7777651

[ref6] BetterUp (2020). The Value of Belonging at Work: Investing in Workplace Inclusion. Available at: https://grow.betterup.com/resources/the-value-of-belonging-at-work-the-business-case-for-investing-in-workplace-inclusion-event (Accessed April 01, 2022).

[ref7] Bispham J. (2006). Rhythm in music: what is it? Who has it? And why? Music. Percept. 24, 125–134. doi: 10.1525/mp.2006.24.2.125

[ref8] Boumaza M. Campana A. (2007). Enquêter en milieu « difficile »: Introduction. Revue Française de Science Politique 57, 5–25. doi: 10.3917/rfsp.571.0005

[ref9] Brewer M. B. Gardner W. (1996). Who is this “we”? Levels of collective identity and self representations. J. Pers. Soc. Psychol. 71, 83–93. doi: 10.1037/0022-3514.71.1.83

[ref10] Chapados C. Levitin D. J. (2008). Cross-modal interactions in the experience of musical performances: physiological correlates. Cognition 108, 639–651. doi: 10.1016/j.cognition.2008.05.008, PMID: 18603233

[ref12] Cloutier J. (2003). Qu’est-ce que l’innovation sociale? Cahier du CRISES. Collection Études thÉoriques. Available at: https://crises.uqam.ca/cahiers/et0314-quest-ce-que-linnovation-sociale/ (Accessed April 01, 2022).

[ref14] Deci E. L. Ryan R. M. (2000). The “what” and “why” of goal pursuits: human needs and the self-determination of behavior. Psychol. Inq. 11, 227–268. doi: 10.1207/S15327965PLI1104_01

[ref15] Deloitte (2020). The Social Enterprise at Work: Paradox as a Path Forward. 2020 Deloitte Global Human Capital Trends. Available at: https://www2.deloitte.com/us/en/insights/focus/human-capital-trends/2020/technology-and-the-social-enterprise.html (Accessed April 01, 2022).

[ref16] DeNora T. (2001). “Aesthetic agency and musical practice: new directions in the sociology of music and emotion,” in Music and Emotion. eds. Juslin P. N. Sloboda J. S. (London: Oxford University Press), 161–180.

[ref18] Dunbar R. (2004). “Language, music, and laughter in evolutionary perspective,” in Evolution of Communication Systems: A Comparative Approach. eds. Oller D. K. Griebel U. (New Jersy: MIT Press), 257–274.

[ref19] Dunbar R. (2012). “On the Evolutionary Function of Song and Dance,” in Music, Language, and Human Evolution. ed. Bannan N. (London: Oxford University Press), 201–214.

[ref20] Dutton J. E. Heaphy E. (2003). “The power of high quality connections,” in Positive Organisational Scholarship: Foundations of a New Discipline. eds. Cameron K. Dutton J. Quinn R. (Oakland: Berrett-Koehler), 263–278.

[ref21] Fox J. G. (1971). Background music and industrial efficiency – A review. Appl. Ergon. 2, 70–73. doi: 10.1016/0003-6870(71)90072-X, PMID: 15676685

[ref22] Frederickson B. (2009). Positivity: Groundbreaking research reveals how to embrace the hidden strength of positive emotions, overcome negativity and thrive. New York: Crown Archetype.

[ref23] Freeman W. J. Wallin N. L. Merker B. Brown S. (2000). “A neurobiological role of music in social bonding,” in The Origins of Music. eds. Wallin N. Merkur B. Brown S. (New Jersy: MIT Press), 411–424.

[ref24] Gallup (2015). State of the global workforce [Report]. Available at: https://www.gallup.com/workplace/238079/state-global-workplace-2017.aspx (Accessed April 01, 2022).

[ref25] Gatewood E. L. (1921). An experiment in the use of music in an architectural drafting room. J. Appl. Psychol. 5, 350–358. doi: 10.1037/h0070493

[ref26] Gaunt H. Duffy C. Coric A. González Delgado I. R. Messas L. Pryimenko O.. (2021). Musicians as “makers in society”: A conceptual Foundation for Contemporary Professional Higher Music Education. Front. Psychol. 12:713648. doi: 10.3389/fpsyg.2021.713648, PMID: 34413817 PMC8368725

[ref28] Groarke J. Hogan M. (2015). Enhancing wellbeing: An emerging model of the adaptive functions of music listening. Psychol. Music 44, 769–791. doi: 10.1177/0305735615591844

[ref29] Haake A. B. (2010). Music listening in offices: balancing internal needs and external considerations. Doctoral Thesis. [Sheffield]: University of Sheffield.

[ref30] Haake A. B. (2011). Individual music listening in workplace settings: An exploratory survey of offices in the UK. Music. Sci. 15, 107–129. doi: 10.1177/1029864911398065

[ref31] Hagen E. H. Bryant G. A. (2003). Music and dance as a coalition signalling system. Hum. Nat. 14, 21–51. doi: 10.1007/s12110-003-1015-z, PMID: 26189987

[ref32] Hargreaves D. J. North A. C. (1999). The functions of music in everyday life: redefining the social in music psychology. Psychol. Music 27, 71–83. doi: 10.1177/0305735699271007

[ref33] Hatzfeld N. (2002). La pause casse-croûte. Quand les chaînes s’arrêtent à Peugeot-Sochaux. Terrain 39, 33–48. doi: 10.4000/terrain.1415

[ref34] Hodson R. (2004). Work life and social fulfilment: does social affiliation at work reflect a carrot or a stick? Soc. Sci. Q. 85, 221–239. doi: 10.1111/j.0038-4941.2004.08502001.x

[ref35] Hove M. J. Risen J. L. (2009). It’s all in the timing: interpersonal synchrony increases affiliation. Soc. Cogn. 27, 949–960. doi: 10.1521/soco.2009.27.6.949

[ref36] Jansson D. Balsnes A. H. (2015). Unfreezing identities: exploring choral singing in the workplace. Int. J. Com. Music 8, 163–178. doi: 10.1386/ijcm.8.2.163_1

[ref01] Kaplan L. Nettel R. (1948). Music in industry. Biol. Hum. Aff. 13, 129–135. 18907595

[ref37] Koelsch S. (2010). Towards a neural basis of music-evoked emotions. Trends Cogn. Sci. 14, 131–137. doi: 10.1016/j.tics.2010.01.002, PMID: 20153242

[ref38] Korczynski M. (2003). Music at work: towards a historical overview. Folk. Music. J. 8, 314–334.

[ref39] Lambert N. M. Stillman T. F. Hicks J. A. Kamble S. Baumeister R. F. Fincham F. D. (2013). To belong is to matter: sense of belonging enhances meaning in life. Personal. Soc. Psychol. Bull. 39, 1418–1427. doi: 10.1177/0146167213499186 23950557

[ref40] Lanza J. (1994). Elevator Music: A Surreal History of Muzak, Easy-Listening and Other Moodsong. New York: St. Martin’s Press.

[ref41] Launay J. Dean R. T. Bailes F. (2013). Synchronization can influence trust following virtual interaction. Exp. Psychol. 60, 53–63. doi: 10.1027/1618-3169/a000173, PMID: 22935329

[ref42] Leary M. R. Kelly K. M. (2009). “Belonging motivation,” in Handbook of Individual Differences in Social Behaviour. eds. Leary M. R. Hoyle R. H. (New York: Guilford), 400–409.

[ref43] Lesiuk T. (2005). The effect of music listening on work performance. Psychol. Music 33, 173–191. doi: 10.1177/0305735605050650

[ref45] Machin A. J. Dunbar R. I. (2011). The brain opioid theory of social attachment: a review of the evidence. Behaviour 148, 985–1025. doi: 10.1163/000579511X596624

[ref47] Maslow A. H. (1954). Motivation and Personality. New York: Harper and Row.

[ref48] Maslow A. H. (1968). Toward a Psychology of Being. 2nd Edn. New York: Van Nostrand.

[ref49] Merker B. H. Madison G. S. Eckerdal P. (2009). On the role and origin of isochrony in human rhythmic entrainment. Cortex 45, 4–17. doi: 10.1016/j.cortex.2008.06.011, PMID: 19046745

[ref52] Moran N. (2014). Social implications arise in embodied music cognition research which can counter musicological “individualism”. Front. Psychol. 5:676. doi: 10.3389/fpsyg.2014.00676, PMID: 25101011 PMC4102907

[ref53] Moynihan D. P. Pandey S. K. (2008). The ties that bind: social networks, person-organization value fit, and turnover intention. J. Public Adm. Res. Theory 18, 205–227. doi: 10.1093/jopart/mum013

[ref54] Nettl B. (2000). “An ethnomusicologist contemplates universals in musical sound and musical culture,” in The Origins of Music. eds. Wallin N. L. Merker B. Brown S. (New York: MIT Press), 463–472.

[ref55] Nolan T. Küpers W. (2009). “Organizational climate, organizational culture, and workplace relationships,” in Friends and Enemies in Organizations. eds. Morrison R. L. Wright S. L. (London: Palgrave Macmillan), 57–77.

[ref57] Oldham G. R. Cummings A. Mischel L. J. Schmidtke J. M. Zhou J. (1995). Listen while you work? Quasi-experimental relations between personal-stereo headset use and employee work response. J. Appl. Psychol. 80, 547–564. doi: 10.1037/0021-9010.80.5.547

[ref58] Park N. Oates S. Schwarzer R. (2013). Christopher Peterson, “other people matter”: 1950–2012. Appl. Psychol. Health Well Being 5, 1–4. doi: 10.1111/aphw.12007, PMID: 23457083

[ref59] Prichard C. Korczynski M. Elmes M. (2007). Music at work: an introduction. Group Org. Manag. 32, 4–21. doi: 10.1177/1059601106294485

[ref60] Raglio A. Oddone E. Morotti L. Khreiwesh Y. Zuddas C. Brusinelli J.. (2020). Music in the workplace: A narrative literature review of intervention studies. J. Compl. Int. Med. 17:20170046. doi: 10.1515/jcim-2017-0046, PMID: 31644428

[ref61] Reddish P. Fischer R. Bulbulia J. (2013). Let’s dance together: synchrony, shared intentionality and cooperation. PLoS One 8:71182. doi: 10.1371/journal.pone.0071182, PMID: 23951106 PMC3737148

[ref62] Reis H. T. Gable S. L. (2003). “Toward a positive psychology of relationships,” in Flourishing: Positive Psychology and the Life Well-Lived. eds. Keyes C. L. M. Haidt J. (United States: American Psychological Association), 129–159.

[ref63] Saliba J. Lorenzo-Seva U. Marco-Pallares J. Tillmann B. Zeitouni A. Lehmann A. (2016). French validation of the Barcelona music reward questionnaire. PeerJ 4:e1760. doi: 10.7717/peerj.1760, PMID: 27019776 PMC4806630

[ref65] Seligman M. E. P. (2011). Flourish: A Visionary New Understanding of Happiness and Wellbeing. United Kingdom: Heinemann.

[ref66] Sloboda J. O’Neill S. A. Ivaldi A. (2001). Functions of music in everyday life: An exploratory study using the experience sampling method. Music. Sci. 5, 9–32. doi: 10.1177/102986490100500102

[ref67] Small C. (1999). Musicking - the meanings of performing and listening. A Lecture. Music Educ. Res. 1, 9–22. doi: 10.1080/1461380990010102

[ref69] Stephens J. P. Heaphy E. D. Dutton J. E. (2011). “High quality connections,” in Handbook of Positive Organizational Scholarship. eds. Cameron K. Spreitzer G. (London: Oxford University Press), 385–399.

[ref70] Thorsén S. (1989). Music Och Arbete: Slutrapport for Projektet Bakgrundsmusik I Arbete Och Fritid (Music and Work: End Report for the Project Background Music at Work and in Leisure), (1–83). Gothenburg: Musicology Department.

[ref71] Tobin S. J. Vanman E. J. Verreynne M. Saeri A. K. (2014). Threats to belonging on Facebook: lurking and ostracism. Soc. Influ. 10, 31–42. doi: 10.1080/15534510.2014.893924

[ref72] Trevarthen C. (2012). “Communicative musicality: The human impulse to create and share music,” in Musical Imaginations: Multidisciplinary Perspectives on Creativity, Performance, and Perception. eds. Hargreaves D. J. Miell D. E. MacDonald R. A. R. (London: Oxford University Press), 259–284.

[ref74] Uhrbrock R. S. (1961). Music on the job: its influence on worker morale and production. Pers. Psychol. 14, 9–38. doi: 10.1111/j.1744-6570.1961.tb00919.x

[ref75] Valdesolo P. DeSteno D. (2011). Synchrony and the social tuning of compassion. Emotion 11, 262–266. doi: 10.1037/a0021302, PMID: 21500895

[ref77] Walton G. M. Brady S. T. (2017). “The many questions of belonging” in Handbook of Competence and Motivation: Theory and Application. eds. Elliot A. Dweck C. Yeager D. (New York: Guilford Press), 272–293.

[ref78] Wiltermuth S. S. Heath C. (2009). Synchrony and cooperation. Psychol. Sci. 20, 1–5. doi: 10.1111/j.1467-9280.2008.02253.x 19152536

