# Supplementary material for: Musicdrops@work: Impact of Shared Listening to Short Live Music Interventions on Sense of Belonging and Subjective Wellbeing at Work
Source: Front Psychol. 2022 Apr 15;13:865938. doi: 10.3389/fpsyg.2022.865938 (PMC9051372; doi:10.3389/fpsyg.2022.865938)
Supplement: Supplementary file 1 [file Table_1.pdf]

Concert	Formation	Repertoire
**1**	Voice and piano	G. Mahler, Ablösung<br>A. Berg, Die Nachtigall<br>G. Rossini, Air of Rosine
**2**	Clarinet and piano	R. Schumann, 2 Fantasiestücke<br>E. Morricone, Mission
**3**	Violoncello and piano	B. Bartók, 3 Hungarian dances<br>C. Saint Säens, The swan
**4**	Voice and piano	A. Dvořák, Air de la lune de Rusalka
**5**	Flute and piano	J. S. Bach, Sicilienne et Badinerie<br>J. Massenet, Meditation from Thaïs
**6**	Violin and viola	B. Martinů, 3 Madrigals<br>C. Gardel, Una cabeza
**7**	Violin and flute	Colombian folk music
**8**	Violin and piano	M. T. Paradis, Sicilienne<br>J. Brahms, 2 Hungarian dances
**9**	Piano 4 hands	C. Debussy, En bateau<br>G. Gershwin, 3 Preludes
**10**	Saxophone and piano	J. Français, 2 Exotic dances<br>A. Piazzola, Etude n° 3
**11**	Oboe, violin and piano	J. S. Bach, Concerto BWV 1060, 1st et 2nd movements

Supplementary Table. Program of musical interventions
